# Supplementary material for: A self-matched leaky-wave antenna for ultrahigh-field magnetic resonance imaging with low specific absorption rate
Source: Nat Commun. 2021 Jan 19;12:455. doi: 10.1038/s41467-020-20708-w (PMC7815766; doi:10.1038/s41467-020-20708-w)
Supplement: Supplementary file 2 — Reporting Summary [file 41467_2020_20708_MOESM2_ESM.pdf]

nature research

Corresponding author(s): Stanislav Glybovski

Last updated by author(s): Nov 23, 2020

Reporting Summary

Nature Research wishes to improve the reproducibility of the work that we publish. This form provides structure for consistency and transparency in reporting. For further information on Nature Research policies, see our Editorial Policies and the Editorial Policy Checklist.

Statistics

For all statistical analyses, confirm that the following items are present in the figure legend, table legend, main text, or Methods section.

n/a	Confirmed	
[x]	[ ]	The exact sample size (n) for each experimental group/condition, given as a discrete number and unit of measurement
[ ]	[x]	A statement on whether measurements were taken from distinct samples or whether the same sample was measured repeatedly
[x]	[ ]	The statistical test(s) used AND whether they are one- or two-sided<br>*Only common tests should be described solely by name; describe more complex techniques in the Methods section.*
[x]	[ ]	A description of all covariates tested
[x]	[ ]	A description of any assumptions or corrections, such as tests of normality and adjustment for multiple comparisons
[ ]	[x]	A full description of the statistical parameters including central tendency (e.g. means) or other basic estimates (e.g. regression coefficient) AND variation (e.g. standard deviation) or associated estimates of uncertainty (e.g. confidence intervals)
[x]	[ ]	For null hypothesis testing, the test statistic (e.g. F, t, r) with confidence intervals, effect sizes, degrees of freedom and P value noted<br>*Give P values as exact values whenever suitable.*
[x]	[ ]	For Bayesian analysis, information on the choice of priors and Markov chain Monte Carlo settings
[x]	[ ]	For hierarchical and complex designs, identification of the appropriate level for tests and full reporting of outcomes
[x]	[ ]	Estimates of effect sizes (e.g. Cohen's d, Pearson's r), indicating how they were calculated

Our web collection on statistics for biologists contains articles on many of the points above.

Software and code

Policy information about availability of computer code

Data collection	Standard PC Interface of Philips MR-platform for MR-images acquisition, CST Microwave Studio 2018, and Empire XPU for numerical electromagnetic simulations.
Data analysis	Dicom Viewer and Matlab 2016 were used for data analysis.

For manuscripts utilizing custom algorithms or software that are central to the research but not yet described in published literature, software must be made available to editors and reviewers. We strongly encourage code deposition in a community repository (e.g. GitHub). See the Nature Research guidelines for submitting code & software for further information.

Data

Policy information about availability of data

All manuscripts must include a data availability statement. This statement should provide the following information, where applicable:

- Accession codes, unique identifiers, or web links for publicly available datasets
- A list of figures that have associated raw data
- A description of any restrictions on data availability

The data that support the presented results are available from the corresponding author upon request.

Field-specific reporting

Life sciences study design

All studies must disclose on these points even when the disclosure is negative.

Sample size	As a sample, we used a pelvis-shaped human torso phantom with electric properties of averaged human body tissues.
Data exclusions	No data were excluded from the analysis.
Replication	The experimental findings' reproducibility was confirmed by in vivo scanning of healthy volunteers and numerical simulation with different human body models.
Randomization	This is not relevant to our study since the data were acquired only from one group.
Blinding	Blinding is not relevant to this study since it was important to demonstrate how the proposed concept works for male volunteer.

Reporting for specific materials, systems and methods

We require information from authors about some types of materials, experimental systems and methods used in many studies. Here, indicate whether each material, system or method listed is relevant to your study. If you are not sure if a list item applies to your research, read the appropriate section before selecting a response.

Materials & experimental systems

n/a	Involved in the study	
☒	☐	Antibodies
☒	☐	Eukaryotic cell lines
☒	☐	Palaeontology and archaeology
☒	☐	Animals and other organisms
☐	☒	Human research participants
☒	☐	Clinical data
☒	☐	Dual use research of concern

Methods

n/a	Involved in the study	
☒	☐	ChIP-seq
☒	☐	Flow cytometry
☒	☐	MRI-based neuroimaging

Human research participants

Policy information about studies involving human research participants

Population characteristics	In-vivo measurments were peformed on single healthy volunteer to prove numerical simulations.
Recruitment	Participant were found among employees of the Department of Physics and Engineering of ITMO University and participated voluntarily.
Ethics oversight	The in-vivo study of a healthy volunteer was approved by the local medical ethics committee of UMC Utrecht (Netherlans) , and informed consent was obtained from the subject.

Note that full information on the approval of the study protocol must also be provided in the manuscript.
